# Reduced Frequency of Cases with Seclusion Is Associated with “Opening the Doors” of a Psychiatric Intensive Care Unit

**DOI:** 10.3389/fpsyt.2018.00057

**Published:** 2018-02-26

**Authors:** Lisa Hochstrasser, Alexander Voulgaris, Julian Möller, Tatjana Zimmermann, Regine Steinauer, Stefan Borgwardt, Undine E. Lang, Christian G. Huber

**Affiliations:** ^1^Universitäre Psychiatrische Kliniken Basel, Universität Basel, Basel, Switzerland; ^2^Abteilung für Psychiatrie und Psychotherapie, Justizvollzugskrankenhaus Berlin, Berlin, Germany

**Keywords:** acute psychiatric treatment, aggression, coercive treatment, closed ward, open doors, compulsory treatment

## Abstract

**Background:**

Implementing an open door policy is a complex intervention comprising changes in therapeutic stance, team processes, and a change from locked to open doors. Recent studies show that it can lead to a reduction of seclusion and forced medication, but the role of the physical change of door status is still unclear.

**Aims:**

The aims of this study is to examine the transition from closed to predominantly open doors on a psychiatric intensive care unit (PICU) and its associations with the frequency of seclusion and forced medication.

**Method:**

A PICU at the Department of Adult Psychiatry, University of Basel, Switzerland, implemented evidence-based strategies for operating an open door policy within the context of acute psychiatry and participated in a hospital-wide implementation of an open door policy before changing door status. 131 inpatient cases hospitalized on this PICU were examined regarding the frequency of seclusion and forced medication using explorative analyses over a time span of 32 weeks (16 weeks after implementation of the new treatment concept but before door opening, 16 weeks after door opening).

**Results:**

Following door status change, the PICU was completely open on 51% of the days and partly open on 23% of the days. The mean number of open hours per day was 12.8 ± 3.9 h. The frequency of forced medication did not change, and the frequency of seclusion decreased significantly [χ^2^ (1, *N* = 131) = 4.73, *p* = 0.036].

**Conclusion:**

This pilot study underlines the potential of a change of door status to attain a reduction in safety measures in the first 4 months.

## Introduction

Psychiatric inpatient wards, for example, in European and Anglo-American psychiatry, are increasingly applying locked door policies (1, 2): locked doors are commonly regarded as an effective measure to deal with safety concerns such as the protection of the public from dangerous persons with psychiatric illnesses (1, 3), protection of patients against the outside, control over patients (4), secure and efficient care, and relief for relatives (5). Furthermore, in clinical practice, it is often assumed that locked doors could also decrease the use of ethically problematic safety measures such as seclusion, restraint, and forced medication (6).

However, contrary to these theoretical advantages of locked door policies, there is increasing evidence that they may not provide higher safety over open door settings regarding suicide, absconding, aggression, and substance use (2, 7–10). In addition, they may unintentionally contribute to an increased use of coercive measures—e.g., a less therapeutic and more authoritarian atmosphere on inpatient units with locked doors has been discussed as being connected with an increased rate of aggressive incidents (4) and could therefore lead to more seclusion, restraint, and forced medication (11). Furthermore, there are several well-known disadvantages of locked door policies (2, 12) like reduced patients’ satisfaction with treatment and care compared to open door policies (13), a worsened therapeutic atmosphere (14, 15), and the patients’ feeling of confinement and dependency (6). Thus, it becomes clear that there is an urgent need to question the current practice of favoring locked door policies.

There is a growing number of recent studies examining the change from locked to open door policies on inpatient wards for acute treatment (16), rehabilitation treatment of psychosis patients (17), treatment of patients with adjustment disorders and personality disorders (18), and a hospital-wide level (11), all showing that this transformation is associated with a reduction of safety measures. However, the introduction of an open door policy constitutes a complex intervention comprising changes in the therapeutic approach toward management of patients at risk for aggression and self-harm, leadership and team processes, and the physical opening of the doors (19), and currently available literature focuses on the summary effect these changes. Although the “open doors” probably contribute to the positive effect of an open door policy, there is therefore currently no literature on the effect of the single intervention of changing from a ward with predominantly closed doors to a policy with predominantly open doors.

### Aim of Study

Therefore, our study aimed at examining the physical introduction of “open doors” on a psychiatric intensive care unit (PICU) where the therapeutic team and the remaining wards of the psychiatric hospital had already undergone the change to an open door policy. We analyzed the association of this intervention with the frequency of safety measures. On the basis of current literature, we hypothesized that the introduction of “open doors” would be associated with a reduced frequency of safety measures in general, seclusion, and forced medication.

## Materials and Methods

### General Framework

This study was carried out at the Department of Adult Psychiatry Basel, University of Basel, Switzerland. In 2010, the hospital comprised 15 psychiatric inpatient wards with a total of 250 beds, of which 6 wards with 114 beds were closed. Beginning in 2011, a newly developed open door policy concept (20, 21) was introduced in a hospital-wide effort. From August 2011 to August 2015, four of the previously closed psychiatric wards were permanently opened. The remaining 2 closed wards were a general PICU with 18 beds admitting persons from 18 to 64 years of age, and an old-age psychiatry ward with 22 beds specialized on management of organic psychiatric disorders and dementia and admitting persons aged 65 years or older.

Regarding the PICU, in a transformation phase from 2012 to May 2015, evidence-based strategies for operating an open door policy within the context of acute psychiatry were applied. These included in particular a systematic change toward a more patient-centered and recovery-oriented care with active family and caregivers involvement, the implementation of a new concept in cognitive behavioral therapy (individual and group therapy), the implementation of a primary nursing care delivery model and de-escalation training for the entire PICU staff. During this process, one-to-one care was increased in crisis situations and management of suicidality and aggression was standardized. After completion of the transformation phase on the May 12, 2015, the PICU was led with the new concept for 16 weeks. Subsequently, on September 1, 2015, the physical door status of the PICU was changed from “locked” to “open whenever possible.”

This study compares the rates of safety measures including seclusion and forced medication between the 16-week period before the door status was changed (doors locked; May 12, 2015, to August 31, 2015) with the 16-week period afterward (open doors whenever possible; September 1, 2015, to December 18, 2015). Notably, the treatment concept of the PICU as well as the composition of the PICU staff including senior physician, clinical psychologists, head nurse, and occupational therapists remained unchanged during the entire study period. PICU staff was aware that sociodemographic and clinical data as well as information on safety measures were routinely documented as a part of the legal obligations of the hospital and that door status was documented to ensure quality management during the transition process, but was not aware of the plans to perform this study until after its completion.

### Study Population

Inclusion criteria for this study were an admission on the PICU at the Department of Adult Psychiatry, Universitäre Psychiatrische Kliniken (UPK) Basel, at least 18 years of age, and admission between the May 12, 2015, and the December 18, 2015. Of the 157 inpatient cases admitted during the observation period, 157 (100%) were entered in the current analyses. No further inclusion or exclusion criteria were defined to ensure a naturalistic sample.

### Documentation and Management of Clinical Data

The door status of the PICU was classified as “fully open” if opened 15 h per day, as “partly open” if opened between 2 and 14 h per day, and as “locked” if opened less than 2 h. The corresponding time data were assessed with a paper-and-pencil diary, in which the PICU staff was asked to record the exact time periods in which the door was locked or open. During the entire study period, the door was closed during the night from 10:00 p.m. until 7:00 a.m.

Clinical and treatment data were continuously documented using the Medfolio software (current version: 2.2.0.2085; NEXUS AG, Villingen-Schwenningen, Germany) and extracted using HCe^®^ Analytics software [Business Intelligence Connector 3 (BIC 3) for patient controlling; TIP Management AG, Dübendorf, Switzerland]. Data on age, gender, nationality, diagnoses according to the International Classification of Diseases, 10th revision [ICD-10 (22)], legal status, type of entry, psychopharmacological treatment, and type of discharge were documented by the psychiatrists responsible for the respective patient. Duration of treatment was calculated from the difference of discharge and admission dates. Legal status was defined as either “voluntary admission” if the patient had consented to inpatient treatment or “involuntary admission.” In Switzerland, legal procedures for involuntary admission differ between cantons; in Basel, only public health officers may initiate an involuntary admission, which leads to one of the lowest rates of involuntarily admitted patients in Basel when compared to other cantons in Switzerland. Legal guardianships are not commonly used in Basel. However, Switzerland allows the “detention in the interest of the patient’s welfare” when “the necessary personal care is otherwise not guaranteed” (23).

Type of discharge was categorized as “both sides agree on discharge,” “discharge without physician’s consent” (i.e., the treating physician would have continued the treatment, but the patient insisted in discharge, and there was no legal basis for continuation of treatment without the patient’s consent), and “discharge without patient’s consent” (i.e., the patient would have liked to continue the treatment, but was discharged nevertheless). Due to legal requirements, a detailed documentation of involuntary treatment was available. Two types of involuntary treatments were recorded: forced isolation with or without psychopharmacological treatment was documented as “seclusion” and forced intake of oral or application of intramuscular medication without forced isolation as “forced medication.” Regarding seclusion, patients were isolated in a room where doors were locked, and a short contact with nurses was practiced every 15 min. Restraint was not practiced in the whole study population as this measure is not used at the UPK. Regarding seclusion and forced medication, the percentage of patients with at least one involuntary measure and the mean number of measures are reported.

As data were documented during routine treatment and anonymized during data extraction, this study was exempt from local ethics committee approval. In addition, the protocol of this study has been peer reviewed by an internal research committee at the Department of Adult Psychiatry, UPK Basel, University of Basel, Switzerland. This study was performed in accordance with all national and international legal regulations and with the Declaration of Helsinki in its current version.

### Statistical Analysis

Clinical and sociodemographic characteristics and the exploratory analysis of the outcome variables “safety measures,” “seclusion,” and “forced medication” are given in total numbers and percentages for nominal scaled variables as well as mean and SD for interval scaled variables. Group comparisons were performed *via* chi-square tests (nominal scale, non-parametric) and one-way ANOVAs (ordinal and interval scale, parametric). Due to the descriptive nature of the exploratory comparisons accompanying and enhancing the sample description (Tables 1 and 2), no correction for multiple testing was employed.

**Table 1:** Clinical and sociodemographic characteristics of cases admitted to the PICU before and after the introduction of predominantly open doors (*N* = 157).

	Closed door period	Open door period	*p* Value
Number of cases	78	79	
Age (years)	39.6 ± 10.9	39.5 ± 11.9	*p* = 0.947^a^
Gender (female)	35 (44.9%)	34 (43.0%)	*p* = 0.873^b^
Nationality			*p* = 0.254^b^
Switzerland	44 (56.4%)	52 (65.8%)	
Other	34 (43.6%)	27 (34.2%)	
**Main diagnosis**			
F0 Organic, including symptomatic, mental disorders	4 (5.1%)	5 (6.3%)	*p* = 1.000^b^
F1 Mental and behavioral disorders due to psychoactive substance use	2 (2.6%)	5 (6.3%)	*p* = 0.442^b^
F2 Schizophrenia, schizotypal, and delusional disorders	48 (61.5%)	39 (49.4%)	*p* = 0.149^b^
F3 Mood (affective) disorders	11 (14.1%)	15 (19.0%)	*p* = 0.520^b^
F4 Neurotic, stress-related and somatoform disorders	5 (6.4%)	7 (8.9%)	*p* = 0.765^b^
F5 Behavioral syndromes associated with physiological disturbances and physical factors	0 (0.0%)	0 (0.0%)	*p* = 1.000^b^
F6 Disorders of adult personality and behavior	5 (6.4%)	4 (5.1%)	*p* = 0.746^b^
F7 Mental retardation	2 (2.6%)	3 (3.8%)	*p* = 1.000^b^
F8 Disorders of psychological development	1 (1.3%)	1 (1.3%)	*p* = 1.000^b^
F9 Behavioral and emotional disorders with onset usually occurring in childhood and adolescence or unspecified mental disorder	0 (0.0%)	0 (0.0%)	*p* = 1.000^b^
No psychiatric disorder	0 (0.0%)	0 (0.0%)	*p* = 1.000^b^
**Type of entry**		
Involuntary	47 (60.3%)	34 (43.0%)	*p* = 0.038^b^
**Type of entry**		
Patient’s initiative	15 (19.2%)	21 (26.6%)	*p* = 0.343^b^
Admission by physician	27 (34.6%)	22 (27.8%)	*p* = 0.393^b^
Other types of admission	36 (46.2%)	32 (40.5%)	*p* = 0.521^b^
Unknown	0 (0.0%)	4 (5.1%)	*p* = 0.120^b^
**Psychopharmacological treatment**		
Antipsychotics	66 (84.6%)	61 (77.2%)	*p* = 0.311^b^
Mood stabilizers	13 (16.7%)	20 (25.3%)	*p* = 0.240^b^
Sedatives	23 (29.5%)	22 (27.8%)	*p* = 0.861^b^
Antidepressants	6 (7.7%)	10 (12.7%)	*p* = 0.430^b^
Treatment duration (days)	24.1 ± 31.7	19.5 ± 23.9	*p* = 0.311^a^
**Type of discharge**		
Both sides agree on discharge	60 (76.9%)	57 (72.2%)	*p* = 0.583^b^
Discharge w/o physician’s consent	9 (11.5%)	14 (17.7%)	*p* = 0.367^b^
Discharge w/o patient’s consent	5 (6.4%)	1 (1.3%)	*p* = 0.117^b^
Other	4 (5.1%)	4 (5.1%)	*p* = 1.000^b^

*Values are given as *n* (%) for nominal variables and in mean ± SD for continuous variables. To enhance the interpretability of the sample description, *p* values from exploratory analyses are presented*.

*^a^One-way ANOVA*.

*^b^χ^2^ test*.

**Table 2:** Clinical and sociodemographic characteristics of cases admitted to inpatient wards of the UPK Basel—except cases admitted to the PICU—before and after the introduction of predominantly open doors on the PICU (*N* = 1,697).

	Closed door period	Open door period	*p* Value
Number of cases	852	845	
Age (years)	46.4 ± 16.6	47.3 ± 17.4	*p* = 0.324^a^
Gender (female)	451 (52.9%)	462 (54.7%)	*p* = 0.495^b^
Nationality			*p* = 0.254^b^
Switzerland	44 (56.4%)	52 (65.8%)	
Other	34 (43.6%)	27 (34.2%)	
**Main diagnosis**		
F0 Organic, including symptomatic, mental disorders	42 (4.9%)	39 (4.6%)	*p* = 0.820^b^
F1 Mental and behavioral disorders due to psychoactive substance use	217 (25.5%)	215 (25.4%)	*p* = 1.000^b^
F2 Schizophrenia, schizotypal, and delusional disorders	138 (16.2%)	140 (16.6%)	*p* = 0.844^b^
F3 Mood (affective) disorders	257 (30.2%)	229 (27.1%)	*p* = 0.179^b^
F4 Neurotic, stress-related, and somatoform disorders	124 (14.6%)	142 (16.8%)	*p* = 0.205^b^
F5 Behavioral syndromes associated with physiological disturbances and physical factors	3 (0.4%)	7 (0.8%)	*p* = 0.223^b^
F6 Disorders of adult personality and behavior	57 (6.7%)	53 (6.3%)	*p* = 0.768^b^
F7 Mental retardation	4 (0.5%)	3 (0.4%)	*p* = 1.000^b^
F8 Disorders of psychological development	0 (0.0%)	5 (0.6%)	*p* = 0.030^b^
F9 Behavioral and emotional disorders with onset usually occurring in childhood and adolescence or unspecified mental disorder	3 (0.4%)	0 (0.0%)	*p* = 0.250^b^
No psychiatric disorder	7 (0.8%)	12 (1.4%)	*p* = 0.259^b^
**Type of entry**			
Involuntary	39 (4.6%)	54 (6.4%)	*p* = 0.110^b^
**Type of entry**			
Patient’s initiative	428 (50.2%)	446 (52.8%)	*p* = 0.308^b^
Admission by physician	330 (38.7%)	293 (34.7%)	*p* = 0.087^b^
Other types of admission	87 (10.2%)	97 (11.5%)	*p* = 0.435^b^
Unknown	7 (0.8%)	9 (1.1%)	*p* = 0.626^b^
**Psychopharmacological treatment**			
Antipsychotics	380 (52.4%)	345 (47.6%)	*p* = 0.117^b^
Mood stabilizers	167 (19.6%)	141 (16.7%)	*p* = 0.131^b^
Sedatives	321 (37.7%)	255 (30.2%)	*p* = 0.001^b^
Antidepressants	394 (46.2%)	391 (46.3%)	*p* = 1.000^b^
Treatment duration (days)	27.5 ± 30.8	25.6 ± 28.9	*p* = 0.192^a^
**Type of discharge**			
Both sides agree on discharge	656 (49.9%)	658 (50.1%)	*p* = 0.685^b^
Discharge w/o physician’s consent	87 (10.2%)	79 (9.3%)	*p* = 0.568^b^
Discharge w/o patient’s consent	24 (2.8%)	31 (3.7%)	*p* = 0.340^b^
Other	85 (10.0%)	77 (9.1%)	*p* = 0.564^b^

*Values are given as n (%) for nominal variables and in mean ± SD for continuous variables. To enhance the interpretability of the sample description, *p* values from exploratory analyses are presented*.

*^a^One-way ANOVA*.

*^b^χ^2^ test*.

All tests of significance were two tailed, and *p* < 0.05 was considered significant. Statistical analyses were conducted using PASW Statistics 18.0 (Chicago, IL, USA).

## Results

After changing the door status from “locked” to “open whenever possible” on September 1, 2015, the examined PICU was fully open on 51% of the days and partly open on 23% of the days within the observation period. The mean number of open hours per day was 12.8 ± 3.9 h. On 26% of the days, the ward was locked.

157 cases were admitted to the PICU during the observation period: 78 cases were admitted before the change of door status and 79 afterward. Table 1 shows the clinical and sociodemographic characteristics of the PICU sample.

Table 2 shows the clinical and sociodemographic characteristics of cases admitted from the May 12, 2015, until the December 18, 2015, to one of the inpatient wards of the UPK Basel except the cases admitted to the PICU.

There were no statistically significant differences between the two subsamples before and after the introduction of predominantly open doors regarding most of the clinical and sociodemographic characteristics in both samples. However, involuntary entries were less frequent in the open door period in the PICU sample. On the other wards, there was a significant albeit clinically irrelevant increase in the frequency of cases with a main diagnosis of ICD-10 chapter F8 from 0 of 852 (0.0%) to 5 of 845 cases (0.6%) and a significant decrease of cases with prescription of sedatives from 321 of 852 (37.7%) to 255 of 845 cases (30.2%).

Table 3 shows the comparison regarding the outcome variables safety measures, seclusion, and forced medication in the PICU sample. All parameters showed a numerical decrease in frequency and the percentage of cases with at least one seclusion decreased significantly [χ^2^ (1, *N* = 131) = 4.73, *p* = 0.036].

**Table 3:** Safety measures before and after the introduction of predominantly open doors on the PICU.

	Closed door period	Open door period	*p* Value
Number of cases	78	79	
**Safety measures**			
Patients with at least one safety measure	23 (29.5%)	13 (16.5%)	0.059^b^
Mean number of safety measures	2.7 ± 2.4	2.3 ± 2.4	0.646^a^
**Seclusion**			
Patients with at least one seclusion	22 (28.2%)	11 (13.9%)	0.032^b^
Mean number of seclusions	2.4 ± 2.3	2.4 ± 2.3	1.000^a^
**Forced medication**			
Patients with at least one forced medication	9 (11.5%)	4 (5.1%)	0.160^b^
Mean number of forced medications	1.1 ± 0.3	1.0 ± 0.0	0.529^a^

*The mean numbers of safety measures, seclusions, and forced medications refer to the cases with at least one safety measure, seclusion, or forced medication, respectively. Values are given as n (%) for nominal variables and in mean ± SD for continuous variables. To enhance interpretability, *p* values from exploratory analyses are presented*.

*^a^One-way ANOVA*.

*^b^χ^2^ test*.

To examine if there was a general decrease of safety measures during the study period, we compared the outcome measures regarding door status on the PICU on a hospital-wide level (see Table 4).

**Table 4:** Safety measures before and after the introduction of predominantly open doors on all wards except the PICU.

	Closed door period	Open door period	*p* Value
Number of cases	852	845	
**Safety measures**			
Patients with at least one safety measure	20 (2.3%)	21 (2.5%)	0.876^b^
Mean number of safety measures	1.4 ± 1.8	2.9 ± 4.2	0.143^a^
**Seclusion**			
Patients with at least one seclusion	12 (1.4%)	13 (1.5%)	0.843^b^
Mean number of seclusions	4.2 ± 2.3	1.8 ± 2.0	0.107^a^
**Forced medication**			
Patients with at least one forced medication	5 (0.6%)	5 (0.6%)	1.000^b^
Mean number of forced medications	1.2 ± 0.5	1.0 ± 0.0	0.347^a^

*The mean numbers of safety measures, seclusions, and forced medications refer to the cases with at least one safety measure, seclusion, or forced medication, respectively. Values are given as n (%) for nominal variables and mean ± SD for continuous variables. To enhance interpretability, *p* values from exploratory analyses are presented*.

*^a^One-way ANOVA*.

*^b^χ^2^ test*.

No significant changes regarding cases with safety measures, seclusion, or forced medication and regarding the mean number of these procedures per affected case could be detected for the other inpatient wards.

## Discussion

This study aimed at examining the effects of the change in door status from locked to predominantly open doors on a PICU that had already undergone transformation to an open door treatment concept on the frequency of safety measures in general, seclusion, and forced medication. The high interference of safety measures and of locked wards with patients’ personal freedom makes this survey highly clinically relevant. Despite the limited time frame of 16 weeks before and after implementing the change, all outcome measures showed a numerical decrease on the PICU, and the decrease in the frequency of seclusions was statistically significant, while there was no hospital-wide decrease of cases affected by seclusions.

To the best of our knowledge, this is the first study that examined the specific associations of the physical change of door status on a PICU with safety measures. Furthermore, PICU staff was not informed about this study until its completion, minimizing effects of potentially present expectations and lowering the chance of a Pygmalion effect (24). In addition, completeness checks and rater trainings according to legal regulations (25) ensured high data quality. Finally, the inclusion and exclusion criteria of this study and the broad spectrum of psychiatric diagnoses of the study population favor inclusion of a naturalistic patient sample and lead to an improved generalizability.

The results concerning the association of door status with safety measures are in accordance with the currently published literature on the implementation of open door policies in a variety of settings (11, 16–18). These findings are frequently challenged by the objection that changes might be simply the effects of a change in patient distribution, e.g., by admitting less severely ill patients after introduction of an open door status, by shifts in patient distribution within a hospital, or by dismissal of agitated or aggressive patients (11). However, it is unlikely that these mechanisms account for the effects seen in our study. As all wards in the hospital apart from a gerontopsychiatric ward were completely open during the observation period, shifts to remaining closed wards were not possible for the PICU patients. Furthermore, the examined hospital is legally obligated to admit all referred patients from its treatment region, preventing selective admission of less severely ill patients. In addition, the concern that the change from a closed door to an open door status might have lead to a premature discharge of patients who are still endangered or dangerous is not supported by our data—there were no significant differences in the treatment duration or in the type of discharge between the study periods. In addition, current literature do not show these effects in other hospitals with an open door policy (8).

A decreased percentage of cases were admitted involuntarily in the open door period. However, it is unlikely that this has biased the results as there were no significant differences in all other examined sociodemographic and clinical characteristics between the locked and open door periods. Furthermore, the decision to admit a person involuntarily can be made only by public health officers, who are not associated with the hospital. Although they could have theoretically be influenced by the change in door status, it is improbable that this would have led to a reduced frequency of involuntary admissions on their behalf because the UPK constitute the only hospital in the treatment area that is legally obligated to admit these patients. A possible explanation compatible with our findings could be a higher patient willingness to be admitted voluntarily after implementation of predominantly open doors.

Some limitations of this study have to be taken into consideration. First, disentangling effects of changes in therapeutic stance, implementation of novel treatment concepts, and team processes from the physical introduction of open doors is challenging, as they are parts of a complex effort to implement an open door policy. Nevertheless, examining a ward that had undergone most of the changes with the exception of introducing predominantly open doors before and after the change in door status constitutes the best possible approximation of the effects of door status change even if there might be additional processes involved. Furthermore, the observational character of the data without comparison group hinders the clear attribution of the decrease in seclusion to the change of door status. Nevertheless, the National Association for Quality Development in Hospitals (ANQ) that monitors the incidence of seclusion for all hospitals in Switzerland found no evidence for a general increase or decrease (25), making it unlikely that the changes are caused by an overall trend to a reduction in safety measures. Furthermore, it would have been advisable to control for multiple admissions of the same patients during the observation periods as this might have potentially biased the results. However, the statistical methods to do so require a larger sample size than available in our study. In addition, the use of routine data prevented the inclusion of factors known to be associated with the frequency of safety measures (e.g., the history of aggression, current psychopathology, and adherence to treatment) as potential moderators in the analysis. Data on physical restraint, defined as mechanical restraint using belts or straps, were not available in this study, as this measure is not used in the examined hospital. In addition, only the initial decision for admission (voluntary or involuntary) is recorded at the UPK Basel. Because data had to be anonymized during extraction, it was not possible to also include information on a possible subsequent retention during the course of hospitalization. Although it would have been of interest to also examine this variable, from clinical experience, the frequency of cases with voluntary entry and subsequent retention is considerably lower than the frequency of cases with initial involuntary admission. Furthermore, patient sample and observation period were limited, and replication studies with greater sample size and longer follow-up are encouraged.

## Conclusion

In this short-time, naturalistic study, the change from locked to predominantly open door status was associated with a statistically significant reduction in the frequency of cases affected by seclusions. This underlines the potential of a door status change to attain a reduction in safety measures.

## Ethics Statement

According to current legal regulation, no approval from the local ethics committee was required for the current study.

## Author Contributions

UL, JM, TZ, and CH designed the study. LH, JM, and RS collected the data. LH, JM, and CH analyzed and interpreted the data. LH, AV, JM, and CH wrote the initial draft of the manuscript. LH had full access to all the data in the study and takes responsibility for the integrity of the data and the accuracy of data analysis. All authors have contributed to, read, and approved the final version of the manuscript. LH and AV contributed equally.

## Conflict of Interest Statement

The authors declare that the research was conducted in the absence of any commercial or financial relationships that could be construed as a potential conflict of interest.

## References

[1] Bowers L Haglund K Muir-Cochrane E Nijman H Simpson A Van Der Merwe M. Locked doors: a survey of patients, staff and visitors. J Psychiatr Ment Health Nurs (2010) 17(10):873–80. 10.1111/j.1365-2850.2010.01614.x 21078002

[2] Schneeberger AR Huber CG Lang UE Open wards in psychiatric clinics and compulsory psychiatric admissions. JAMA Psychiatry (2016) 73(12):1293 10.1001/jamapsychiatry.2016.1738 27829088

[3] Sowislo JF Gonet-Wirz F Borgwardt S Lang UE Huber CG. Perceived dangerousness as related to psychiatric symptoms and psychiatric service use – a Vignette Based Representative Population Survey. Sci Rep (2017) 8:45716. 10.1038/srep45716 28367993 PMC5377934

[4] Bowers L Jarrett M Clark N. Absconding: a literature review. J Psychiatr Ment Health Nurs (1998) 5(5):343–53. 10.1046/j.1365-2850.1998.00149.x 10067481

[5] Haglund K von Knorring L von Essen L Psychiatric wards with locked doors – advantages and disadvantages according to nurses and mental health nurse assistants. J Clin Nurs (2006) 15(4):387–94. 10.1111/j.1365-2702.2006.01489.x 16553751

[6] Haglund K von Essen L Locked entrance doors at psychiatric wards – advantages and disadvantages according to voluntarily admitted patients. Nord J Psychiatry (2005) 59(6):511–5. 10.1080/08039480500360781 16316906

[7] Lang UE Hartmann S Schulz-Hartmann S Gudlowski Y Ricken R Munk I Do locked doors in psychiatric hospitals prevent patients from absconding? Eur J Psychiat (2010) 24(4):199–204. 10.4321/S0213-61632010000400001

[8] Huber CG Schneeberger AR Kowalinski E Frohlich D von Felten S Walter M Suicide risk and absconding in psychiatric hospitals with and without open door policies: a 15 year, observational study. Lancet Psychiatry (2016) 3(9):842–9. 10.1016/S2215-0366(16)30168-7 27477886

[9] Schneeberger AR Kowalinski E Frohlich D Schroder K von Felten S Zinkler M Aggression and violence in psychiatric hospitals with and without open door policies: a 15-year naturalistic observational study. J Psychiatr Res (2017) 95:189–95. 10.1016/j.jpsychires.2017.08.017 28866330

[10] Steinauer R Huber CG Petitjean S Wiesbeck GA Dursteler KM Lang UE Effect of door-locking policy on inpatient treatment of substance use and dual disorders. Eur Addict Res (2017) 23(2):87–96. 10.1159/000458757 28351023

[11] Jungfer HA Schneeberger AR Borgwardt S Walter M Vogel M Gairing SK Reduction of seclusion on a hospital-wide level: successful implementation of a less restrictive policy. J Psychiatr Res (2014) 54:94–9. 10.1016/j.jpsychires.2014.03.020 24726637

[12] Lang UE Walter M Borgwardt S Heinz A About the reduction of compulsory measures by an “Open Door Policy”. Psychiatr Prax (2016) 43(6):299–301. 10.1055/s-0042-111032 27607432

[13] Muller MJ Schlosser R Kapp-Steen G Schanz B Benkert O. Patients’ satisfaction with psychiatric treatment: comparison between an open and a closed ward. Psychiatr Q (2002) 73(2):93–107. 10.1023/A:1015099526445 12025725

[14] Blaesi S Gairing SK Walter M Lang UE Huber CG Safety, therapeutic hold, and patient’s cohesion on closed, recently opened, and open psychiatric wards. Psychiatr Prax (2015) 42(2):76–81. 10.1055/s-0033-1359871 24254421

[15] Lo SB Gaupp R Huber C Schneeberger A Garic G Voulgaris A Influence of an "Open Door Policy" on ward climate: impact on treatment quality. Psychiatr Prax (2017). 10.1055/s-0042-121784 28371949

[16] Cibis ML Wackerhagen C Muller S Lang UE Schmidt Y Heinz A Comparison of aggressive behavior, compulsory medication and absconding behavior between open and closed door policy in an acute psychiatric ward. Psychiatr Prax (2017) 44(3):141–7. 10.1055/s-0042-105181 27399589

[17] Fan Z Huang J Wu Q Jiang S. Comparison of standard locked-ward treatment versus open-ward rehabilitation treatment for chronic schizophrenic patients. A one-year controlled trial in Canton. Br J Psychiatry Suppl (1994) 165(24):45–51. 7946231

[18] Steinert T Eisele F Goeser U Tschoeke S Uhlmann C Schmid P. Successful interventions on an organisational level to reduce violence and coercive interventions in in-patients with adjustment disorders and personality disorders. Clin Pract Epidemiol Ment Health (2008) 4:27. 10.1186/1745-0179-4-27 19014698 PMC2596103

[19] Sharfstein SS Commentary: reducing restraint and seclusion: a view from the trenches. Psychiatr Serv (2008) 59(2):197 10.1176/appi.ps.59.2.197 18245164

[20] Sollberger D Lang UE Psychiatry with open doors: part 1: rational for an open door for acute psychiatry. Nervenarzt (2014) 85(3):312–8. 10.1007/s00115-013-3769-9 23538944

[21] Sollberger D Lang UE Psychiatry with open doors: part 2: therapeutic challenges. Nervenarzt (2014) 85(3):319–25. 10.1007/s00115-013-3770-3 23579876

[22] WHO. ICD-10: The ICD-10 Classification of Mental and Behavioural Disorders: Clinical Descriptions and Diagnostic Guidelines. Geneva: World Health Organisation (WHO) (1992).

[23] Riecher-Rossler A Rossler W Compulsory admission of psychiatric patients – an international comparison. Acta Psychiatr Scand (1993) 87(4):231–6. 10.1111/j.1600-0447.1993.tb03363.x 8488742

[24] Rosenthal R Critiquing pygmalion: a 25-year perspective. Curr Dir Psychol Sci (1995) 4(6):171–2. 10.1111/1467-8721.Ep10772607

[25] Nationaler Verein für Qualitätsentwicklung in Spitälern und Kliniken (ANQ). Nationaler Vergleichsbericht Stationäre Psychiatrie Erwachsene. (2013, 2014). Available from: http://www.anq.ch/messergebnisse/ergebnisse-psychiatrie

